# Three years pilot of spinal muscular atrophy newborn screening turned into official program in Southern Belgium

**DOI:** 10.1038/s41598-021-99496-2

**Published:** 2021-10-07

**Authors:** François Boemer, Jean-Hubert Caberg, Pablo Beckers, Vinciane Dideberg, Samantha di Fiore, Vincent Bours, Sandrine Marie, Joseph Dewulf, Lionel Marcelis, Nicolas Deconinck, Aurore Daron, Laura Blasco-Perez, Eduardo Tizzano, Mickaël Hiligsmann, Jacques Lombet, Tatiana Pereira, Lucia Lopez-Granados, Sarvnaz Shalchian-Tehran, Véronique van Assche, Arabelle Willems, Sofie Huybrechts, Bénédicte Mast, Rudolf van Olden, Tamara Dangouloff, Laurent Servais

**Affiliations:** 1 Biochemical Genetics Laboratory, Human Genetics, CHU Sart-Tilman, University of Liège, B35, 4000 Liège, Belgium; 2 Molecular Genetics Lab, Department of Human Genetics, CHU of Liège, University of Liège, Liège, Belgium; 3 Department of Human Genetics, CHU of Liège, University of Liège, Liège, Belgium; 4 Department of Laboratory Medicine, Cliniques Universitaires Saint-Luc, UCLouvain, Brussels, Belgium; 5 Laboratory of Pediatric Research, Free University of Brussels, Brussels, Belgium; 6 Neuromuscular Reference Center, Hôpital des Enfants Reine Fabiola (HUDERF), Université Libre de Bruxelles, Brussels, Belgium; 7 Division of Child Neurology, Reference Center for Neuromuscular Diseases, Department of Pediatrics, CHR Citadelle, University of Liège, Liège, Belgium; 8 Department of Clinical and Molecular Genetics, Hospital Valle Hebron, Barcelona, Spain; 9 Department of Health Services Research, CAPHRI Care and Public Health Research Institute, Maastricht University, Maastricht, The Netherlands; 10 ONE, Office de la Naissance et de l’Enfance, Herstal, Belgium; 11 Department of Neurology and Ethic Committee, CHR Citadelle, Liège, Belgium; 12 ABMM, Association Belge contre les Maladies neuro-Musculaires ASBL, La Louvière, Belgium; 13 Biogen, Cambridge, MA USA; 14 Roche, Basel, Switzerland; 15 Novartis Gene Therapies Switzerland GmBH, Zürich, Switzerland; 16 MDUK Neuromuscular Center, Department of Paediatrics, University of Oxford, Oxford, UK

**Keywords:** Motor neuron disease, Population screening

## Abstract

Three new therapies for spinal muscular atrophy (SMA) have been approved by the United States Food and Drug Administration and the European Medicines Agency since 2016. Although these new therapies improve the quality of life of patients who are symptomatic at first treatment, administration before the onset of symptoms is significantly more effective. As a consequence, newborn screening programs have been initiated in several countries. In 2018, we launched a 3-year pilot program to screen newborns for SMA in the Belgian region of Liège. This program was rapidly expanding to all of Southern Belgium, a region of approximately 55,000 births annually. During the pilot program, 136,339 neonates were tested for deletion of exon 7 of *SMN1*, the most common cause of SMA. Nine SMA cases with homozygous deletion were identified through this screen. Another patient was identified after presenting with symptoms and was shown to be heterozygous for the *SMN1* exon 7 deletion and a point mutation on the opposite allele. These ten patients were treated. The pilot program has now successfully transitioned into the official neonatal screening program in Southern Belgium. The lessons learned during implementation of this pilot program are reported.

## Introduction

Spinal muscular atrophy (SMA) is a neuromuscular disorder characterized by muscle atrophy resulting from the degeneration of motor neurons in the spinal cord. SMA is caused by biallelic pathogenic variants in the *SMN1* gene, which encodes Survival of Motor Neuron (SMN), a protein essential for survival of motor neurons^1^. Approximately 95% of patients carry a homozygous deletion of exon 7 in the *SMN1* gene, the remaining 5% of cases are due to the deletion of exon 7 on one allele and a deleterious variant on the opposite allele. *SMN2* is a pseudogene that differs from *SMN1* by only a few nucleotides, including a C to T transition in exon 7. This variant results in the skipping of exon 7 in about 90% of *SMN2* transcripts, thereby encoding a truncated, unstable protein. The full-length, functional SMN protein results from approximately 10% of *SMN2* transcripts. The number of *SMN2* copies is inversely correlated with the severity of the phenotype. Patients with two copies usually present with the most severe and frequent form of spinal muscular atrophy, SMA1. In these patients, symptom onset usually occurs before the age of 6 months, and this type of SMA is associated with high mortality and morbidity^2^. Patients with a larger number of copies of *SMN2* may present with symptoms long after acquisition of ambulation; a limited few even develop symptoms in adulthood. Currently, SMA is classified into four types, SMA1, SMA2, SMA3, and SMA4, based on maximal motor ability achieved.

Over the last few years, several new treatments for SMA have dramatically improved the prognosis of affected patients^3^. Nusinersen^4^ was the first drug to be approved by the United States Food and Drug Administration (FDA) and the European Medicines Agency (EMA) in December 2016 and June 2017, respectively. In Belgium, nusinersen has been reimbursed by the healthcare system since September 2018. More recently, onasemnogene abeparvovec-xioi gene therapy^5^ also received FDA and EMA approval, in May 2019 and May 2020 respectively. The marketing authorization of a third drug, risdiplam^6^, was granted by the FDA last year, and it also received a positive opinion from the EMA’s Committee for Medicinal Products for Human Use (CHMP) in February 2021. Several other drugs are currently in development^7^.

Based on these recent advances in SMA management and on evidence showing that patients treated presymptomatically have better outcomes^8,9^, newborn screening (NBS) for SMA has begun in several countries^10–18^. Moreover, in 2018 SMA was included in the Recommended Uniform Screening Panel (RUSP), the list of disorders that the US Department of Health and Human Services recommends be screened for as part of NBS programs^19^.

In early 2018, the authors of this paper and Neuromuscular Reference Centers (NMRCs) of Southern Belgium launched a 3-year NBS pilot program for SMA under the project title “Sun May Arise on SMA”. The pilot project was done in close collaboration with our industry partners AveXis, Biogen, and Roche, who funded a significant part of the program, as well as with the governmental agency in charge of NBS in Southern Belgium, the Office of Birth and Childhood (Office de la Naissance et de l’Enfance, ONE)^20,21^. It should be noted that NBS is not a federal competency in Belgium, and therefore such initiatives are conducted by a separate government agency in Northern Belgium.

The initial pilot phase of the ‘Sun May Arise on SMA’ project transitioned into an official program in Southern Belgium on 1 March 2021. Northern Belgium has correspondingly made a political commitment to include SMA in their official program in 2022.

This manuscript reports the key insights gained during the pilot effort.

## Results

### Inclusion of SMA in the NBS program

The process that led to implementation of the NBS program for SMA in Southern Belgium has been previously reported^20^. A key principle was involvement of all stakeholders from the beginning. Political, ethical, and clinical partners, including genetic and screening labs, were involved in the project’s governance.

### Incidence

**Figure 1:**
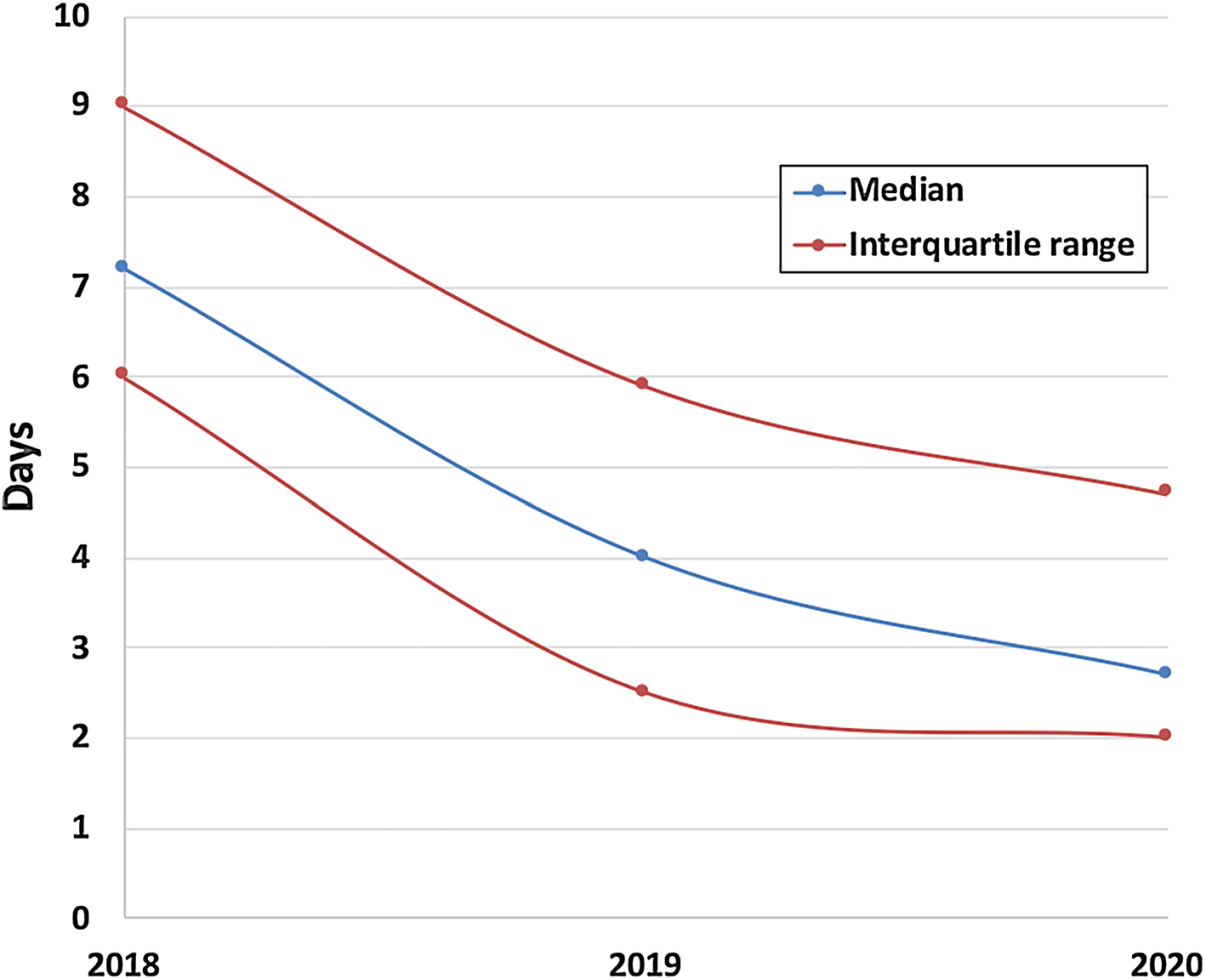
TAT improvement over the study period.

Over the 3-year pilot study from March 2018 to February 2021, 136,339 neonates were tested for the *SMN1* exon 7 deletion using a previously described qRT-PCR test with fluorescence read-out^20^. The dispersion plot of the ratio of *SMN1* to the housekeeping gene *RPP30* allowed clear discrimination between positive (i.e., SMA patients with a homozygous deletion of exon 7) and negative results (Fig. 1).

Nine SMA cases were identified. To our knowledge, no newborn carrying a homozygous deletion was missed over this period. All patients with symptoms of neuromuscular disease in Belgium are referred to an NMRC, thus it is quite unlikely that such a case could happen without one of the centers being informed. Nevertheless, we cannot rule out the possibility that a patient with SMA3 or SMA4 born during the period of the pilot study may be diagnosed in the future.

One SMA1 patient was not be diagnosed through NBS. The neonate was heterozygous for the *SMN1* exon 7 deletion and had the c.815A>G (p.Tyr272Cys) point mutation on the opposite allele. This patient was referred to an NMRC at the age of 4 months, after the onset of symptoms compatible with SMA.

This corresponds to an incidence for SMA in Southern Belgium of 1 in 13,634 newborns (95% confidence interval: 1/8417 to 1/35,858). The incidence of homozygous deletion is 1 in 15,149 individuals (95% confidence interval: 1/9163 to 1/43,696).

### Neonate referral

Positive screening results were immediately communicated by the laboratory to both the neonate’s pediatrician and to referent neurologists in NMRC. The parents were contacted on the same day by a referent neuro-pediatrician or by a pediatrician of the maternity ward and consultation was planned as soon as possible. Thanks to the second-tier MLPA testing performed on DBS-extracted DNA, the number of *SMN2* copies was available to the clinician at the patient’s first visit, and therefore the clinician could immediately explain relevant therapeutic options to parents. The neonate’s blood was then drawn to perform the MLPA confirmatory analysis. There were no false positives from the initial DBS testing.

**Figure 2:**
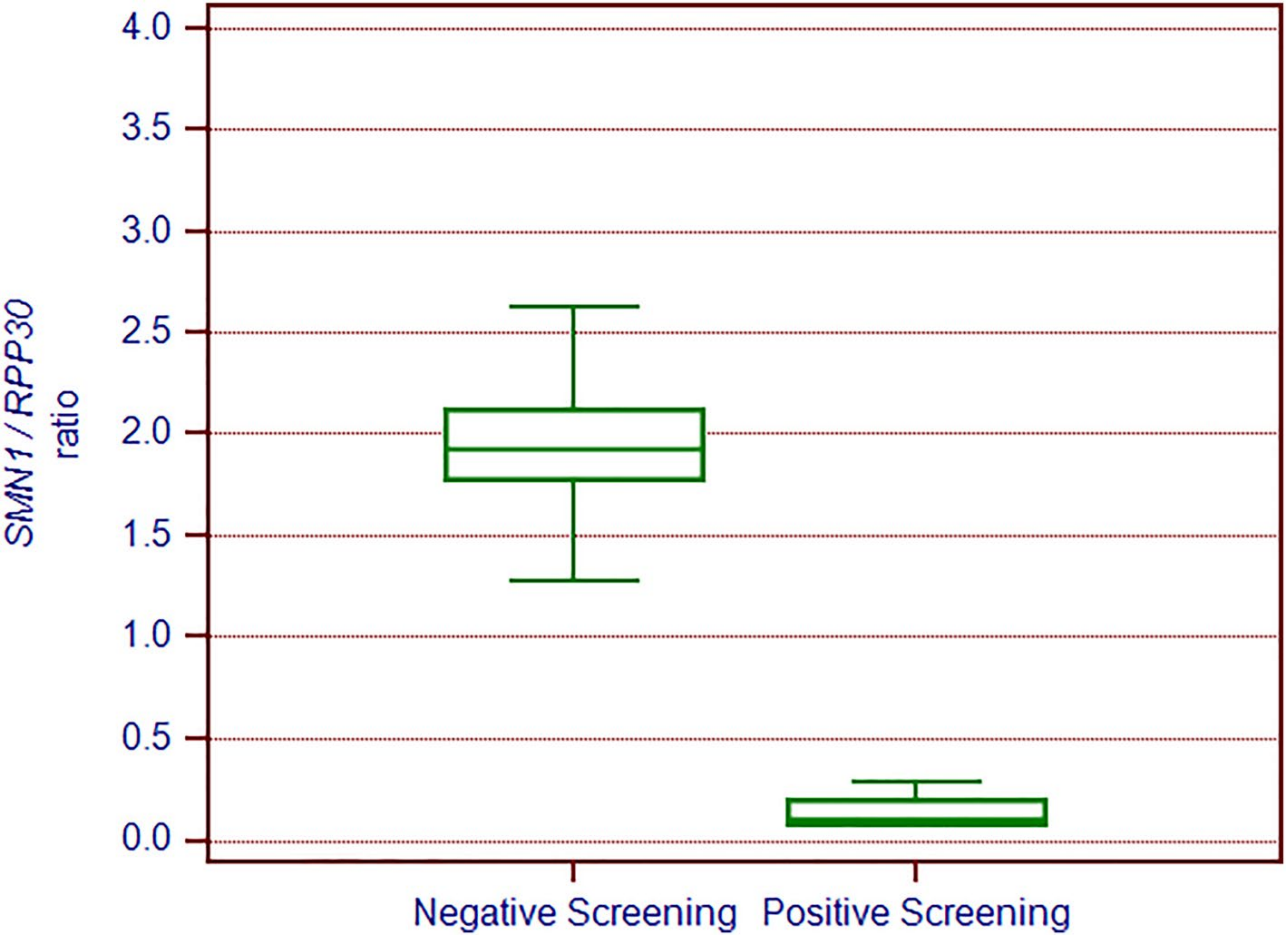
Box-and-whisker plot of the endpoint-fluorescence *SMN1* to *RPP30* ratio for negative (n = 136.330) and positive (n = 9) screening results.

The screening and diagnostic timelines for the ten SMA patients are detailed in Table 1. All nine patients identified through NBS began treatment before the age of 2 months. In order to ensure the most efficient management of patients, it is important to save time. Over the course of the project, the turnaround time (TAT) was considerably improved. For the first 9 months, the population coverage was limited to Liège NBS center, where about 300–350 samples were analyzed each week. The median TAT, calculated for the interval between DBS reception in Liège’s center and validation of the result, was 7.2 days (interquartile range: 6.0–9.0 days). At the beginning of 2019, the other two NBS centers in Southern Belgium joined the project, outsourcing their analytical process to Liège’s center, and the number of samples analyzed increased to approximately 1200 samples per week. Early in 2019, acquisition of a dedicated qPCR instrument and hiring of a devoted lab technician permitted a considerable scale-up of our analytical throughput. Subsequently, TAT was reduced from 7.2 days in 2018 (interquartile range: 6.0–9.0 days) to 4.0 days later in 2019 (interquartile range: 2.5–5.9 days) and to 2.7 days in 2020 (interquartile range: 2.0–4.7 days) (Fig. 2).

**Table 1:** Screening and diagnostic timeline (in post-natal days) for SMA patients identified by NBS.

ID	DBS sampling	DBS received by NBS center	DBS received by Liège lab	First-tier results	Second-tier results	Parents contacted	First visit	Treatment initiation	Delay between first visit and treatment initiation
1	3	4	4	11	18	20	21	32	11
2	3	8	8	27	30	30	31	38	7
3	4	5	9	13	13	13	14	41	27
4	4	13	19	27	27	31	32	54	22
5	4	9	29	31	35	35	37	49	12
6	3	4	11	18	22	20	21	39	18
7	3	7	15	17	21	18	20	29	9
8	3	5	15	18	19	22	23	32	9
9	3	6	6	9	10	9	10	30	20
Median	3	6	11	18	21	20	21	38	12

### Patient treatment and outcomes

Parents were informed about the different therapeutic options during first visit. Nusinersen was available in Belgium from the start of the study. Risdiplam and the gene therapy onasemnogene abeparvovec-xioi were not commercially available in the country during the pilot study but were accessible through several concurrent clinical trials in NMRC (Spr1nt: NCT03505099, STRIVE-EU: NCT03461289, Rainbowfish: NCT03779334). For the six patients who received nusinersen, treatment began an average of 10 days after the first consultation (7–20). Parents of Patient 9 initially refused the treatment, which explains the delay in initiation. The delay between the first consultation and the initiation of treatment was the longest for the three patients who participated in the therapeutic trials (18, 22, and 27 days) as participation in a trial required testing prior to inclusion. Patients who showed early clinical manifestations of the disease, even if weak (i.e., only areflexia), were those who had two copies of *SMN2*. These patients had developmental delays despite treatment. Patients with three or four copies of *SMN2* showed no symptoms at the time of treatment initiation and hit motor developmental milestones at the usual ages. *SMN2* copy number and modifier variants, treatment regimen, and evolution of symptoms in identified patients are summarized in Table 2.

**Table 2:** *SMN2* copy number and polymorphisms, treatment, and evolution of symptoms of SMA patients identified during the study period.

Id	Sex	*SMN2* copy number	*SMN2 polymorphism*		Treatment	Treatment initiation in days	Phenotype at treatment start	Sitter (in months)	Walker (in months)	Age at last assessment (in months)	Max score on CHOP-INTEND scale^c^	Max score on HINE 2 scale^d^
			c.859G > C	c.835‐44A > G								
1	M	3	Negative	Negative	Nusinersen	32	Asymptomatic	7	13	33	64	26
2	F	2	Negative	Negative	Nusinersen	38	Areflexia, discrete hypotonia,	7	27 with help	32	58	24
3	M	3	Negative	Negative	OA^b^	41	Asymptomatic	7	15	24	64	24
4	M	2	/	/	OA^b^	54	Discrete hypotonia	6,5	Stand up alone	22	51	20
5	M	4	Negative	Negative	Nusinersen	49	Asymptomatic	6	12	22	64	–
6	F	4	Negative	Negative	Risdiplam	39	Asymptomatic	5	12	20	64	26
7	M	2	Negative	Negative	Nusinersen	29	Areflexia	6	No	18	60	17
8	M	2	Negative	Negative	Nusinersen	32	Areflexia	6	No	14	54	–
9	F	3	Negative	Negative	Nusinersen	30	Asymptomatic	7	11	12	62	21
10^a^	M	2	/	/	Nusinersen	150	Proximal hypotonia, areflexia, tongue fasciculations	No	No	17	34	2

^a^Compound heterozygous patient identified at the age of 4 months.

^b^Onasemnogene abeparvovec-xioi.

^c^CHOP-INTEND maximum score is 64.

^d^HINE Sect. “Results” maximum score is 26.

A dash indicates that the test was not given.

### Lessons learned from individual cases

#### The case of treatment refusal

The parents of one patient initially refused treatment. The child had three copies of *SMN2* and was asymptomatic at the time of diagnosis. The parents were not French speakers, and at the initial consultation were accompanied by a French-speaking cousin serving as a translator. This was not an optimal situation, as the translator was emotionally invested and only partially translated the physician’s explanation to the parents. Following their refusal, they were offered a second consultation with two different child neurologists and a psychologist with a professional translator in attendance, and a further consultation was also proposed with a German-speaking neurologist. The parents stated several times that they would prefer to wait for their daughter to present with symptoms before discussing treatment. This prompted internal discussions among the clinical team to balance the right of parents to make decisions regarding the care of their child with the rights of the child given that clinical evidence clearly indicates that treatment before symptom onset is necessary to ensure the possibility of normal development^8,9^.

After requesting several external medical and external opinions, we explained to the parents that the clinical team could not carry the responsibility of withholding care, and that the family court would have to be consulted. After receiving initial opinions from the prosecutor supportive of intervention, the parents accepted the necessity of treatment. Interestingly, the relationship between the clinical care team and the family remained positive, and 1 year after birth the mother stated that they had been in such an emotional state that they were ‘unable to make the right decision’ and now recognized that treatment was the best solution.

No other parents refused treatment. Some parents indicated their preference for a particular treatment. The choice to proceed with a treatment was always made in light of treatment availability, the child's clinical condition, and the scientific data available at the time, and with the mutual agreement of the treating physicians and the parents.

#### Patients and siblings with four copies of SMN2

As mentioned earlier, treatment of children is specifically discussed with the parents. In the two cases with four copies of *SMN2* identified during the pilot study, the parents promptly agreed to the proposal to initiate early treatment.

One of the patients identified with four copies of *SMN2* had two older siblings, aged 4 years and 6 years and 6 months, respectively. Interestingly, the mother presented with two copies of *SMN1* and the father with one copy. We then discovered that the maternal grandmother had three copies of *SMN1*, two on the same chromosome, and the paternal grandmother had only one copy. The mother was 2/0, which means that she would not have been identified as at-risk during carrier testing.

The initial clinical examination of the siblings of the patient indicated normal development, but the parents wished to have them tested. This was done, and we found that, like the infant, both children had the homozygous deletion of exon 7 of *SMN2* and four copies of *SMN2*. Their parents opted to delay treatment. Further evaluations of the siblings were performed after 3 months.

The physician had concerns regarding the potential muscle weakness of the older sibling, but the parents again opted to delay treatment. When the child was aged 7 years and 4 months, a video sent by the parents clearly confirmed a proximal weakness and fatigability. On examination, there was an absence of patellar reflex, and the need for the child to support himself with a hand on his leg when rising from the floor. The motor function measure and six-minute walk test were stable. The parents refused to treat at this stage.

At 7 years and 11 months, the electromyography (EMG) showed a 30% loss of motor amplitude. At 8 years, the same difficulties at the clinical examination were noticed with a complete absence of reflexes, and unchanged compound muscle action potential.

The second sibling, who was 4 years old at the time of diagnosis, showed no deficit in either the clinical examination, physiological tests or EMG. Follow-up is continuing with clinical and physiotherapy examinations every 6 months. To date, at the age of 5 years and 6 months, the second child is still wholly asymptomatic.

### Transition to health authorities: a strong partnership among stakeholders

Retrospectively, the key element in the successful transition from the trial project to a government-sanctioned public health program was the involvement and unanimous support of all stakeholders from the beginning of the project and throughout its duration. Transitioning to an official program was an initial objective of the pilot program. The involvement of patient advocacy groups, neuromuscular reference centers, and newborn screening centers, as well as public engagement through broadcast and social media (such as on the study’s Facebook page, www.facebook.com/sunmayariseonsma) also significantly facilitated the rapid and smooth transition to an official program.

A clear governance structure helped to build a strong partnership between pilot study leaders, the regional agency in charge of NBS, and NBS centers. Public involvement gave rise to support from across the political spectrum in Belgium. The ordinance incorporating SMA into the NBS list for Southern Belgium was passed by the Parliament of Wallonia on 4 February 2021 for implementation on 1 March 2021, with immediate handover from the study team to the public health service after the completion of the 3-year pilot project. UCLouvain and ULBruxelles NBS centers are incorporating the SMA screening test into their own infrastructure.

## Discussion

The incidence of SMA of 1 in 15,149 determined during the NBS pilot study in Southern Belgium is broadly consistent with previous studies. The incidence reported in Taiwan was 1 in 17,181 neonates^12^. In Germany, 30 SMA cases were identified during screening of 213,279 DBS cards for a incidence of 1 in 7109 infants^17,22^. Australian NBS has identified nine SMA patients in 103,903 newborns screened for an incidence of 1 per 11,544^18^. New York State recently screened more than 225,000 neonates and reported a much lower incidence of 1 per 28,137^23^. The authors of that study argued that the low SMA incidence reported in their area is likely due to biased estimates, coupled with increased awareness and access to carrier screening, genetic counselling, cascade testing, prenatal diagnosis, and advanced reproductive technologies. A better understanding of this low incidence is of primary importance since it could have consequences on reimbursement for disease-modifying therapies and NBS funding decisions^24^.

Surprisingly, we did not identify any SMA neonates during the third year of our pilot study. Based on the Poisson distribution of rare events, the probability of diagnosing no cases of SMA over 1 year is 2.5% (Table 3). Given the low probability that there should be no cases in a year, we hypothesized that carrier screening and prenatal testing had contributed to this outcome. We therefore contacted various molecular genetics centers in Southern Belgium to request the number of positive results for SMA based on pre-conceptional and prenatal diagnosis during the corresponding period. However, they reported no positive results that could explain this absence of cases over the previous year. Subsequently, three new cases were identified in the first 4 months following the end of the pilot, which further reinforces the hypothesis of a pure random distribution.

**Table 3:** Poisson probability of case occurrence in Southern Belgium based on annual periods.

Screening period	03/2018–02/2019	03/2019–02/2020	03/2020–02/2021
Number of screened newborns	22,930	57,607	55,802
Expected number of SMA cases (λ)	1.51	3.80	3.68
Probability of 0 cases during period	0.220	0.022	**0.025**
Probability of 3 cases during period	**0.127**	0.204	0.209
Probability of 6 cases during period	0.004	**0.094**	0.087

Bold values correspond to the number of SMA cases actually identified during the designated period.

Our study is, to our knowledge, the first to report a SMA patient compound heterozygous for the *SMN1* exon 7 deletion and a point mutation on the opposite allele, in the context of NBS. Because the first-tier assays specifically target the homozygous *SMN1* deletion, this patient was not be identified during the screening process. Rather, the patient was identified at the age of 4 months, after referral for mild hypotonia. The clinical sensitivity of SMA NBS is estimated between 95 and 98%, as affected individuals who are compound heterozygotes (i.e., those with one *SMN1* allele lacking exon 7 and a point mutation on the second allele) are missed^11,25^. To date, no false negatives or false positives have been identified in our screening program.

The five neonates with either three or four copies of *SMN2* were all asymptomatic at treatment start (Table 2). Most presented the highest Children's Hospital of Philadelphia Infant Test of Neuromuscular Disorders (CHOP-INTEND) and Hammersmith Infant Neurologic Examination, Sect. “Results” (HINE-2) scores during their last motor assessment (age range: 12–33 months). The four newborns with two copies of *SMN2* showed a slight hypotonia and/or a discrete areflexia when the treatment was initiated. These patients did not get the highest scores on CHOP-INTEND and HINE-2 scales during their last motor assessment (age range: 14–32 months). Of these four patients, three were treated with the approved nusinersen therapy. Treatment initiation may thus be considered as relatively delayed (range: 29–54 days) when compared to first visit (range: 20–32 days). This lag may be a factor that has impaired the most favorable outcome for these patients. In the future, we hope that the recent transition of our pilot study into the official neonatal screening program will facilitate a more prompt care.

The overall evidence for the efficacy of early treatment of patients with SMA has been recently reviewed^26^. It is likely that the cost of the new SMA treatments initially hampered the implementation of NBS programs by the political authorities. Presently, the substantial cost burden of standard care for patients with SMA is estimated to be between US$ 75,047 and US$ 196,429 per year for SMA1 patients, and between US$ 27,157 and US$ 82,474 for other types of SMA^27^. Therefore, given the high cost-to-benefit ratio of drugs approved at current prices when administered to post-symptomatic patients^27^, we know it is critical to identify patients prior to symptom onset. A medico-economic evaluation with assessment of patient quality of life is also currently ongoing to assess the cost-effectiveness of our NBS program^20^. Pre-treatment levels of phosphorylated neurofilaments are a validated marker of nerve cell damage in pre-symptomatic and in young SMA1 patients^28^. These levels decrease exponentially in pre-symptomatic SMA patients with two *SMN2* copies, indicating acute and severe neuronal loss^9^. These data indicate that it is critical to begin treatment of SMA1 patients with as little delay as possible. An NBS program is accordingly an ideal method for early identification of these infants.

There were several incidents encountered during this pilot program, the description of which may help other NBS programs more effectively communicate with the parents of recently diagnosed infants.

In one case, parents initially refused treatment. In hindsight, this might have been avoided if a professional translator had been present during the first consultation. In another case, three SMA-affected children of a mother with two copies of *SMN1* on the same allele were diagnosed as a result of NBS: the youngest through the NBS pilot program itself and his siblings following this initial positive identification. As the mother would not have been identified as at-risk during carrier testing, this clearly indicates that carrier screening should not be relied upon as the sole strategy against SMA.

Finally, we were faced with a case of a patient with symptoms that the parents refused to recognize. Political authorities must therefore put plans in place to deal with cases of refusal of treatment. Presently, some countries leave the decision of treatment to a multidisciplinary consultation meeting, whereas others leave all choice to the parents. The present authors believe that the interest of the child must take priority over parents' rights. A collegial discussion of these potential issues prior to implementation of an NBS program is necessary.

Our study suffers from the small size of the studied population. Southern Belgium has a total population of approximately 4.5 million people; therefore the number of cases identified in the neonate population remains low.

Today, nine countries around the world have started SMA NBS, with the number of newborns screened set to increase in the coming years as further countries embark on similar programs^29^. Our project confirms that a pilot program can be rapidly transitioned into the official NBS program. Given the effective treatments now available for SMA and the importance of treatment prior to the onset of symptoms, testing for SMA should be incorporated into screening of all newborns.

## Materials and methods

### Newborn samples

NBS samples were collected on Whatman^®^ 903 cards between 48 and 120 h of life either in maternity wards or at home, in accordance with legal requirements of the federal authority (Wallonie–Bruxelles Federation) in charge of NBS in Southern Belgium.

The dried blood spot (DBS) cards were sent to selected neonatal screening laboratories. No additional sampling was required to incorporate SMA testing in the standard NBS panel as the residual blood spots collected for conventional NBS were sufficient to test for SMA. After analysis, filter papers are stored at room temperature for 5 years.

As detailed in our previous manuscript^20^, parental consent was not required for participation in this study. While strongly recommended, NBS is not mandatory in Southern Belgium and parents are informed that they have the right to refuse screening for their child. This opt-out option is not disease-specific; it applies to the neonatal screening panel as a whole. The project was approved by our ethical review board (reference number B412201734396), in accordance with the Declaration of Helsinki.

### NBS assay and confirmatory method

**Figure 3:**
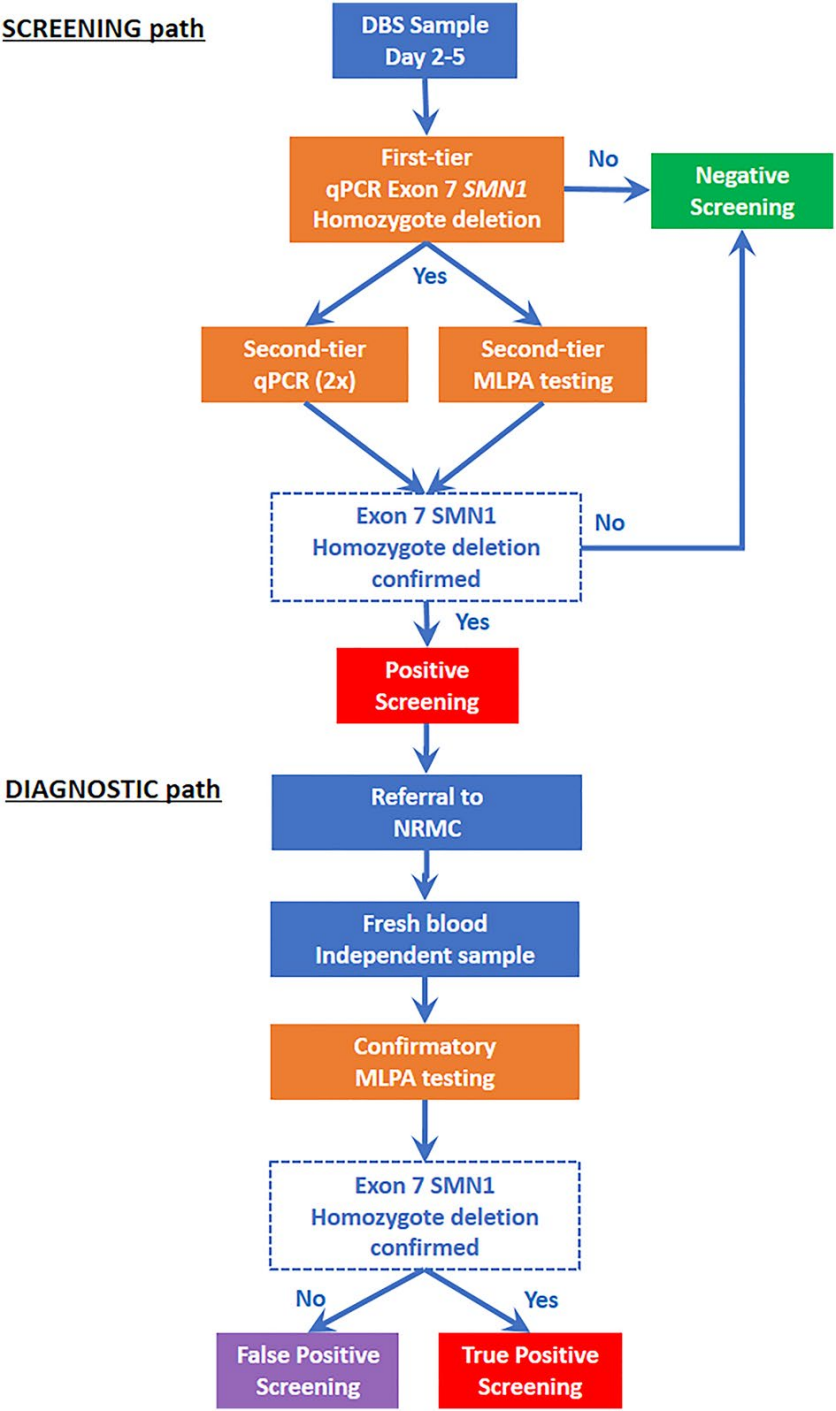
Screening and diagnostic flowchart.

The flow chart for screening for SMA is shown in Fig. 3. We designed a quantitative polymerase chain reaction (qPCR) assay to specifically detect homozygous deletions of *SMN1* exon 7 on DNA extracted from DBS^20^. DNA extraction was performed by alkaline denaturation at 98 °C. qPCR amplification was performed in 96-well plates, preloaded with primers, dye-labeled probes, and master mix provided by Eurogentec. This assay cannot identify heterozygous carriers of the deletion of exon 7 or *SMN1* point mutations, and the number of copies of *SMN2* were not determined in this first-tier assay. Given the importance of *SMN2* copy number in SMA management, qPCR-positive results were confirmed by the multiplex ligation-dependent probe amplification (MLPA) technique, which also provided information on *SMN2* status. For this purpose, we used the Salsa MLPA Probemix P021 SMA diagnostic kit (MRC Holland).

First-tier positive samples were re-analyzed twice from the same DBS. Simultaneously, a second-tier MLPA assay was performed from the same DNA extracted for the first-tier qPCR. Upon positive results from confirmatory testing, neonates were immediately referred to a neuro-pediatrician in one of the NMRCs involved in the trial. At the first visit, fresh blood was collected to confirm the positive screening result by MLPA on an independent sample. Additionally, we also sequenced the *SMN2* gene to look for the presence of both c.859G>C and c.835‐44A>G intragenic modifier variants. A *SMN2*-specific PCR has been used to amplify exons 7 and 8 and study the presence or absence of the positive modifier variants. The primers (available on request) were designed based on the paralogous sequence variants described by Blasco-Pérez et al.^30^, in order to achieve specificity towards *SMN2* (Blasco-Perez et al., in preparation).

### Population coverage

There are approximately 55,000 annual births in Southern Belgium, and NBS for these infants is carried out by three independent academic centers. The current project was launched in March 2018 in Liège’s NBS laboratory, which screens about 16,000 newborns per year. Due to strong support from the supervisory authorities and the efforts of the project management team to promote the project, the pilot study rapidly expanded to include the two other screening centers of Southern Belgium, UCLouvain and ULBruxelles. In order to rapidly implement the program in these two centers, DNA was extracted in the lab to which the DBS card was sent. Sealed microtiter plates containing samples for SMA screening were then transferred to the lab in Liège, which ran qPCR assays on all samples. SMA screening was offered to the entire neonate population of Southern Belgium beginning in early 2019.

### Clinical and therapeutic protocol

All patients were examined by board certified neuro-paediatricians with expertise in SMA. The different therapeutic options were proposed to parents during the first visit. The phenotype at the start of treatment and the ages of sitting and walking acquisitions were recorded. Longitudinal motor milestone assessment was evaluated by trained physiotherapists, using CHOP-INTEND and HINE-2 scales.

### Statistical analyses

Exact probability of rare event occurrence was estimated by a Poisson distribution in which the probability mass function is p(x) = e^−λ^·λ^x^/x!, where λ is the average number of events per year, and x is number of events in each interval.

### Ethics approval

Ethical approval (reference B412201734396) was obtained from the Institutional Review Board (Ethical Committee of the Hospital CHR Citadelle, Liège, Belgium) in compliance with the Declaration of Helsinki.

## Data Availability

The data that support the findings of this study are available from the corresponding author, FB, upon reasonable request.

## Abbreviations

ABMM: Association Belge contre les Maladies neuro-Musculaires
CHMP: Committee for Medicinal Products for Human Use
CHOP-INTEND: Children's Hospital of Philadelphia Infant Test of Neuromuscular Disorders
DBS: Dried blood spot
EMA: European Medicines Agency
EMG: Electromyography
ERB: Ethical review board
FDA: Food and Drug Administration
HINE: Hammersmith Infant Neurologic Examination
MLPA: Multiplex ligation-dependent probe amplification
NBS: Newborn screening
NMRC: Neuro Muscular Reference Centers
ONE: Office de la Naissance et de l’Enfance
qPCR: Quantitative polymerase chain reaction
RUSP: Recommended Uniform Screening Panel
SMA: Spinal muscular atrophy
SMN: Survival of Motor Neuron
TAT: Turnaround time
